# Coronary Artery Calcium Score: A Review

**DOI:** 10.5812/ircmj.16616

**Published:** 2013-12-05

**Authors:** Abbas Arjmand Shabestari

**Affiliations:** 1 Radiology Department, Modarres Hospital, Shahid Beheshti University of Medical Sciences, Tehran, IR Iran; 2 Advanced Diagnostic and Interventional Radiology Research Center (ADIR), Tehran, IR Iran

**Keywords:** Atherosclerosis, Tomography, X-Ray Computed, Arteries, Coronary Artery Disease, Calcium

## Abstract

**Context:**

Coronary artery disease (CAD) is the foremost cause of death in many countries and hence, its early diagnosis is usually concerned as a major healthcare priority. Coronary artery calcium scoring (CACS) using either electron beam computed tomography (EBCT) or multislice computed tomography (MSCT) has been applied for more than 20 years to provide an early CAD diagnosis in clinical routine practice. Moreover, its association with other body organs has been a matter of vast research.

**Evidence Acquisition:**

In this review article, techniques of CACS using EBCT and MSCT scanners as well as clinical and research indications of CACS are searched from PubMed, ISI Web of Science, Google Scholar and Scopus databases in a time period between late 1970s through July 2013 and following appropriate selection, dealt with. Moreover, the previous and ongoing research subjects and their results are discussed.

**Results:**

The CACS is vastly applied in early detection of CAD and in many other research fields.

**Conclusions:**

CACS has remarkably changed the screening techniques to detect CAD earlier than before and is generally accepted as a standard of reference for determination of risk of further cardiac events.

## 1. Context

Coronary artery disease (CAD) is the foremost cause of death in many countries throughout the entire world. Albeit more common in Western countries, it seems to be increasing in frequency through the non-industrialized countries, as well, likely reflecting a change in their inhabitants’ lifestyles (1).

Over the past decades it has been well demonstrated that coronary artery atherosclerotic plaques are the main causes of CAD. While in progression, the coronary artery plaques may contain calcium; hence, it has been suggested that finding of calcified foci at coronary artery walls may indicate CAD and its extent. From the early 1980s the presence of coronary arterial mural calcified foci was found to be related to CAD (2-4).

Indeed, Margolis et al. (5) showed the significance of coronary artery calcification in diagnosis of CAD and in determining its prognosis. In their study the calcified foci were detected in coronary arteries area at fluoroscopic assessment of 800 patients and their impact on future cardiac events was evaluated (5, 6).

For the first time in 1979 Guthaner and her colleagues demonstrated the ability of computed tomography (CT) to find coronary artery calcifications (7, 8). Thereafter, it was gradually demonstrated that CT is much more sensitive than fluoroscopy in calcium detection (9, 10). On the other hand, beating heart has always been a problem for imaging, requiring faster image acquisition to improve the temporal resolution, so electron beam CT (EBCT) scanners also known as ultrafast CT scanners were further developed. CT scanner developments resulted in ability to find smaller coronary calcified foci enabling the researchers to make reproducible quantitative measurements in coronary artery calcium scoring (CACS) (11-13).

## 2. Evidence Acquisition

The terms “coronary”, “calcium”, “score” and “computed tomography” were searched among the databases of PubMed, ISI Web of science, Scopus and Google Scholar to find relevant data from late 1970s (introduction of body CT) through July 2013 and totally 1023 published resources were found. Irrelevant and repetitive resources (n = 926) were excluded based on their titles and/or Abstract contents and a few (n = 8) others (comprising unpublished ones until then) were included in study. Ultimately, 105 references were assessed.

## 3. Results

### 3.1. Technical Aspects

The EBCT scanners provided electron ray targeting a ring anode around the patient’s body and hence, obviated the need for presence of a mechanically-rotating X-ray tube, resulting in remarkably faster image acquisition and thereby, improving the temporal resolution (11, 12, 14). For the first time EBCT which could provide appropriate temporal resolution for cardiac imaging was used for CACS (15, 16). However, EBCT was not applicable in imaging of most other body organs, since its high noise - low signal-to-noise ratio (SNR) - resulted in poor image quality, while its improved temporal resolution had not any positive impact on imaging of other non-moving or less-moving body organs. The aforementioned high image noise leads to inappropriate image quality and hence, EBCT is usually not used in coronary CT-angiography (CCTA). Moreover, EBCT is more expensive than most routinely used CT scanners and its dimensions are larger, requiring remarkably more roomy space for scanner to be installed. Using EBCT the parameters for CACS were as follows: longitudinal (z-axis) scan coverage from tracheal carina to diaphragm during breath hold, no contrast agent injection, 3 mm slice thickness, electrocardiography (ECG)-gated sequence (non-spiral) mode acquisition at 80% of R-R interval, 512 X 512 matrix size, 130 kVp, current of 630 mA and 100 mSec acquisition time (15). As pointed out before, the EBCT scanners were merely applied to determine CACS. Introducing multislice CT (MSCT) scanners from 1998 and their substantial developments through the last 15 years has led to their gradual supersession of the EBCT (17). Given the remarkably better spatial resolution of MSCT scanners, nowadays ECG-synchronized CCTA is widely used and in most cases, CACS is performed as a conjunct and usually prior to CCTA using MSCT (18-21). The advancements of MSCT scanners led to improvement of spatial resolution and SNR as well as less noisy images in comparison with EBCT. From their introduction, the temporal resolution of various MSCT generations has gradually improved from 500 mSec in first 4-slice MSCT scanners to less than 100 mSec in recently introduced ones (22). The improved imaging quality has led to extended use of MSCT in CCTA. The same holds true about CACS and hence, nowadays MSCT is much more widely used than EBCT for CACS (23). A high image quality is obtained using MSCT scanners when utilizing retrospective ECG-gating technique to find the most appropriate phase with least motion artifact (24, 25). Moreover, Horiguchi et al have shown that using prospective ECG-triggered scan at 45% of R-R interval in a 64-slice MSCT scanner provides a constantly high image quality irrespective of heart rate, body mass index or background noise level (26). In a study by Groen et al both 64-slice MSCT scanner and dual-source CT scanner were compared with EBCT, revealing more association of dual-source CT with EBCT in comparison with 64-slice MSCT. This higher correlation was more perceptible when using thinner slices and particularly 0.6 mm slices (27). It had been shown by Ulzheimer and Kalender (28) that image quality of 4-slice MSCT scanners for CACS was equal or even better than EBCT for cardiac imaging. Horiguchi et al found a high agreement between 16-slice MSCT scanner and EBCT in CACS (29). The inter-scan variability was demonstrated to be less than EBCT when performed by MSCT scanners in a study by Kopp et al. (30). Assessment of CACS using thin-slice (0.5 mm) 320-slice scanner by Van der Bijl (31) showed that small calcified plaques detection is more accurate when compared with standard thicker (3 mm) slices (31). When compared with standard 3 mm thickness EBCT technique, various both thicker and thinner slices were applied to determine CACS. Thick-slice (5-6 mm) EBCT scan was used by Detrano et al. (32) in order to shorten scanning time and reduce the background noise, which demonstrated similar scores and prognostic value in CAD. On the other hand, there are a few studies that showed applicability of thinner than 3 mm slices using MSCT scanners (which reveal less noise than EBCT images) in CACS (27, 31, 33). Not only the slice thickness is of paramount importance, but also the reconstruction interval of slices has its impact on CACS. The effect of using varying reconstruction intervals in 16-slice MSCT scanner is assessed by Schlosser et al. (34); while recently, it has been demonstrated to have a significant importance in CACS accuracy using dual-source CT scanners, particularly in cases of low calcium score (24). Frequent use of CACS protocol as a routine adjunct of CCTA leads to increase of patient’s radiation dose. The radiation dose associated with CAC scoring is small and ranges from 0.9 to 2.4 milliSievert (mSv) in different multislice CT scanners (35). In some cardiac CT protocols, the radiation doses are estimated to be higher than 10 mSv (35-37) leading to a small but measurable increase in the risk of radiation-induced cancer and hence this fact should be concerned in cases that CACS and repeated exams are used as a widespread population screening (36). Some have proposed that while CCTA is able to assess the CAD extent, CACS may not be necessary to be carried out; nevertheless, it has been shown that whenever the density of intraluminal contrast is increased, coronary mural calcified plaques may not be detected and hence, resultant from their similar attenuation values may be missed (31). Image densities are shown to be variable based on CT scanner type as well as patient’s body habitus by Nelson et al and using calibration phantoms has been demonstrated to reduce the inter-scan variability in calcium density measurements (38). An automatic attenuation-based tube current adaptation technique was proposed by Mühlenbruch et al in a 16-slice MSCT scanner to reduce patient’s radiation dose and image noise in CACS (39).

### 3.2. Agatston Score

**Figure 1.**
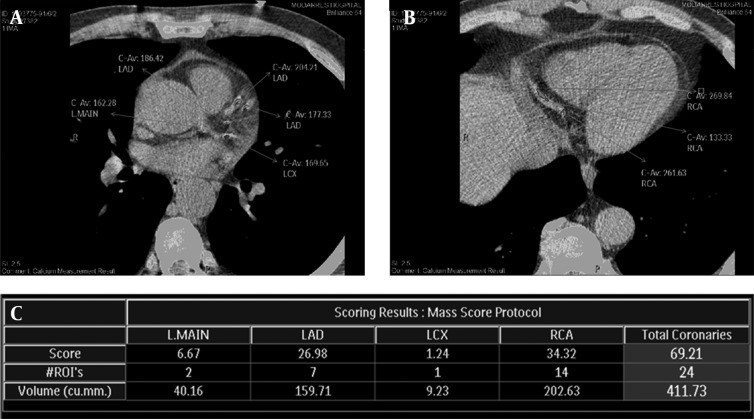
Coronary calcium score non-contrasted ECG-gated computed tomographic (CT) views of coronary arteries demonstrate presence of multiple calcified plaques through the anatomic territory of left main (L.MAIN) coronary artery and proximal segments of left anterior descending (LAD) and left circumflex (LCX) coronary arteries and their branches (A) and distal segment of right coronary artery (RCA) (B). The measurement table (C) provided by CT workstation demonstrates the calcium score of each coronary artery and their total score based on Agatston technique in the first row, the number of assigned calcified plaques in each territory and their total number in the second row and measured area of the corresponding plaques (according to square millimeters) in the third row. The measured total coronary calcium score (389.57 Agatston Units) in this 66-year old man equals to 77% for that particular gender (male) and age range (65-69 years) according to an available database calculated and shown in the last row.

The first practically applicable quantitative CACS protocol was introduced by Arthur Agatston and his colleagues ( 15 )in 1990 and has still remained the standard method in CACS. Any structure which had densities of 130 Hounsfield units (HU) or more and having an area of 1 mm2 or more was segmented as calcified focus and those foci overlying the anatomic site of coronary arteries were considered to represent calcified plaques. Using an area of at least 1 mm^2 ^- comprising of at least 2 pixels - ensured one not including single pixel - which represents image noise - in measurements. In each segmented calcified focus, based on its peak density, a density score of 1 through 4 was assigned. The stratified density scores 1, 2, 3 and 4 represented the highest densities 130-199 HU, 200-299 HU, 300-399 HU and ≥ 400 HU, respectively. The most important determining factors in calculating calcium score of each plaque were the measured area of each calcified plaque and its density. The total Agatston score (AS) of each individual was calculated by summing the scores of every calcified focus through all of the coronary arteries (15). Figure 1 shows the Agatston score measurement technique and its resultant values demonstrated in a dedicated table. 

### 3.3. Volume Score

Since AS required a relatively complex measurement technique, in an effort to simplify the coronary calcium measurement and increase its reproducibility, “volume score” was first introduced by Callister et al. (40) simply calculated based on segmented calcified plaque area and number of slices containing each of those plaques. The volume score was expressed in milliliters. No peak density measurement is used in volume score calculation and hence, its inter-scan variation is less than AS. Nonetheless, this variability increases in high calcium scores, so Hokanson et al. (41) used the square root of volume score in order to decrease this variation. Analysis of AS and volume score has been shown to be similar in reference data establishment of age-sex percentile ranking (42, 43).

### 3.4. Mass Score

**Figure 2.**
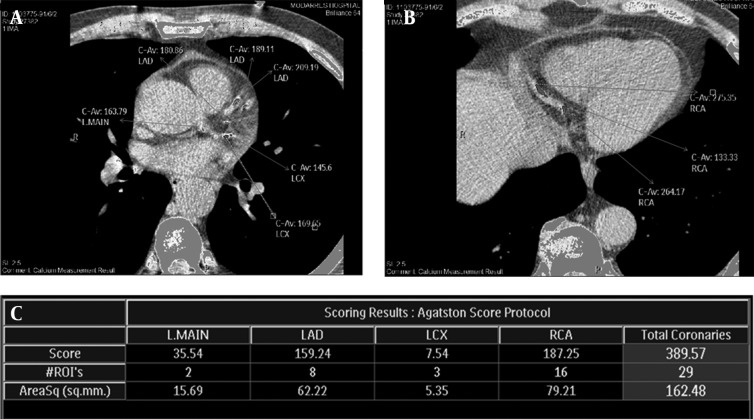
Coronary calcium score non-contrasted ECG-gated computed tomographic (CT) views of coronary arteries of the same patient as in Fig-1 demonstrating presence of multiple calcified plaques through the anatomic territory of left main (L.MAIN) coronary artery and proximal segments of left anterior descending (LAD) and left circumflex (LCX) coronary arteries and their branches (A) and distal segment of right coronary artery (RCA) (B). The measurement table (C) provided by CT workstation demonstrates the mass calcium score of each coronary artery and their total score based on Mass Score protocol in the first row, the number of assigned calcified plaques in each territory and their total number in the second row and measured area of the corresponding plaques (according to square millimeters) in the third row. The total coronary calcium Mass Score is measured to be 69.21 in this individual.

In 2002 Hong et al. (33) introduced a technique to measure “mass score” of calcified coronary plaques which measures the absolute real mass of coronary calcium. Albeit it may be considered more accurate and more reproducible than Agatston and volume scores, it requires a phantom containing different concentrations of calcium hydroxyapatite (CaHA) placed beneath the patient’s thorax in order to calibrate the segmented coronary calcium and hence, is more complicated than former ones in hardware (33, 44). The absolute score is expressed as milligrams of CaHA in this stratification. Despite presence of a few papers revealing its weaknesses, it is shown that mass score may be even more reproducible than Agatston or volume scores in high scores, so that Ulzheimer and Kalender in 2003 (28) suggested changing from a particular Hounsfield Unit threshold to a calcium equivalent expressed as mg of CaHA/cm^3^ to calculate CACS. Based on their suggestion, this new measurement would not differ among varying CT scanners (28). Figure 2 demonstrates the mass score measurement technique and corresponding calculated values shown in a dedicated table.

There is no published absolute quantification reference standard of plaque burden. Nevertheless, standard for risk stratification in percentile for Agatston, volume and mass scores was published by Rumberger and Kaufman in 2003 (42) as a large-scale (in 11,490 patients) research. The mass score is considered to be a reliable CACS technique both in research and in clinical routine practice.

### 3.5. Calcium Coverage Score

More recently in 2008, Brown et al. (45) established another technique called calcium coverage score. Applying calcium coverage score in a Multi-Ethnic Study of Atherosclerosis (MESA) associated with its correlation with AS and mass score, the coronary arteries percentage involved in calcification was called calcium coverage score. Calcium coverage score was shown to be accompanied by hypertension and diabetes as well as dyslipidemia (45).

### 3.6. Validation and Clinical Applications of CACS

Many studies were carried out based on Agatston method trying to use this technique to stratify patients’ CAD extent, one of the first ones being Rumberger’s study. Rumberger and his colleagues provided a stratified guideline according to CACS in order to determine the coronary plaque burden and resultant CAD severity shown in Table 1 (46). Hoff et al. (16) used the same EBCT technique in more than 35,000 individuals to determine the AS percentiles in each gender and each of the categorized age groups. Another study based on the same age-gender percentiles have shown the significantly higher CAD risk of those above the 75th percentile in comparison with those below the 25th percentile (47). In 2003, Rumberger & Kaufman (42) using Agatston, mass and volume CACS techniques suggested another risk stratification model for CAD in more than 11,000 individuals. 

**Table 1.** The first Rumberger guideline based on Agatston score using Electron Beam Computed Tomography (EBCT) ( 47 )

Calcium Score	Plaque Burden	Clinical Interpretation
**0**	None	Very low risk of cardiovascular disease
		Likelihood of coronary artery disease presence <5%
		Negative examination
**1-10**	Minimal	Significant coronary artery disease very unlikely
**11-100**	Mild	Likely mild or minimal coronary stenosis
**101-400**	Moderate	Moderate non-obstructive coronary artery disease highly likely
**Over 400**	Extensive	High likelihood of at least one significant coronary stenosis (> 50% diameter)

Over the past years CACS validity has been assessed in many studies. Since it was previously shown that there is poor correlation between the EBCT findings of coronary calcium and the luminal narrowing severity in coronary catheter angiography, Rumberger et al. (48) made a comparison of EBCT measured calcified atherosclerotic plaque area with plaque area measured in histopathologic findings of 13 heart autopsy exams. They figured out that there was a close association between plaque extent and coronary calcification area. The discrepancies could be described by the presence of non-calcified plaques and the so called “positive remodeling” of coronary arteries (48).

Guerci et al. (49) showed that the AS has a remarkably significant correlation with coronary narrowing severity and therefore, suggested that CACS could represent CAD extent. Rumberger and his colleagues (50) presented a CACS cut-off point to predict the severe luminal narrowing. They proposed AS of 371 as predictor of more than 70% narrowing in as a minimum one of the coronary arteries. Similarly, Moser et al suggested 400 AS could be considered an edge score for a further requirement to carry out nuclear myocardial perfusion scan in asymptomatic patients (51).

According to Mendoza-Rodriguez and colleagues using 64-slice MSCT, volume score was significantly correlated with flow-limiting CAD (52).

Shaw and colleagues assessed coronary calcium as a risk in all-cause mortality estimate and included 10,377 asymptomatic subjects proving that calcification provides non-dependent information additionally to Framingham risk factors (53).

Comparing with prevalence of CAD in 17,967 asymptomatic individuals, Cheng et al. (54) realized that there was an increased risk of CAD at all levels higher than 95 AS. Guerci and colleagues in a study of of 290 subjects suggested 80 as the cutoff AS value in forecasting the augmented CAD likelihood. Based on these findings, the absolute AS value revealed its potential as a sensitive method for CAD screening (55). Using MSCT, Shabestari et al showed a moderate-to-good agreement between CACS of more than 100 AS and significant coronary stenosis (56). Through the recent years the clinical clarification of a ‘‘zero’’ score has been subject of major debate. Wexler et al. (57) described that a zero calcium score almost with certainty implied CAD absence. Nevertheless, it should be reminded that absence of coronary calcified plaque does not exclude presence of soft plaque (and resultant acute coronary syndrome). Shemesh et al. (58) declared that there was a contradiction as minimal CACS could characterize those who may present with acute symptoms, whereas presence of diffusely distributed and high-density calcified plaques could be associated with chronic coronary events. Thompson and Stanford commented that the zero calcium might exclude significant narrowing but could not rule out the CAD and suggested that if there is not any risk factor, there won’t be any requirement for further diagnostic procedures (59). Knez et al. (60) demonstrated that calcified plaque absence could very precisely exclude significant CAD in individuals in an age group higher than 50 years. On the other hand, Ergun et al showed that there were a substantial amount of subjects who had zero CACS and their CCTA revealed CAD (61). Actually, importance of absent calcified plaque is currently considered to be weaker than that before. Sometimes clinically important soft plaques were detected in CCTA of patients who had no coronary calcium. Nonetheless, Uretsky et al. (62) reported that non-calcified plaques were hardly accompanied by significant stenoses, if ever. Church and colleagues (63) proposed that absence of calcium demonstrates a remarkably limited CAD likelihood. As Grayburn has pointed out in his article, it is important to assess the CAC score in the clinical context before further tests are recommended for patients (64). The well-known Framingham risk score enables prediction of cardiac events in asymptomatic individuals and is estimated according to age, gender, total serum cholesterol level, high-density lipoprotein (HDL) cholesterol level, history of smoking, and systolic blood pressure. This score is denoted as 10-year risk score for the estimation of CAD events likelihood. Nonetheless, growing evidences exist which demonstrate these risk stratification techniques have significant limitations as guidance for treatment of each individual. Based on the question of whether CACS is indicated for screening asymptomatic patients at Framingham intermediate risk for CAD the guidelines differ, however, CACS for symptomatic patients with known CAD is generally accepted to be not helpful. The main aim of CACS in asymptomatic persons is to refine the risk assessment to determine whether preventive measures have to be intensified, not finding persons with symptomatic coronary stenosis (23). In those people who are asymptomatic, absence of calcified plaque is accompanied by a remarkably mild (< 1% in each year) risk of main coronary events through the upcoming 3-5 years, while presence of high CACS in asymptomatic individuals may increase this risk up to 11-fold (65). A MESA paper revealed that there is a remarkable variation in CACS measured in various ethnicities. Nevertheless, CACS had an additive importance in their prognosis determination so that in those people who had AS > 100, in comparison with those who had not any calcified plaque, the prevalence of coronary events may show a 7-fold increase (66). CACS is not recommended for screening of individuals who are symptomatic as calcified plaques only have marginal relation to the extent of narrowing and the significance of absent or low CACS in symptomatic patients remains unclear (21). Symptomatic patients should be referred for CCTA to determine the CAD severity and there is no significant incremental value of CACS beyond the CCTA prognostic information in symptomatic patients (67).

Controversies regarding the clinical interpretation and application of “zero CACS” in different risk-stratified cohorts of patients have continued to persist and are summarized in Table 2. There are some publications which suggest applying the calcium coverage score as a useful filter for CCTA to diagnose noteworthy coronary disease in individuals who present as having chest discomfort. These imply that zero CACS can exclude obstructive coronary disease and hence obviate the requirement of any further assessment using imaging, while those individuals who have higher CACS may further undergo CCTA to determine their stenosis severity. Nonetheless, it should be pointed out that CACS and coronary CTA can be under remarkable influence of CAD pretest likelihood: severe CACS foresees significant CAD while concurrently degrades the CCTA image - because of blooming artifact of calcium - so that CCTA may be excluded in the management of patients with high CACS (68, 69). Meng and coworkers (69) demonstrated that a score of more than 400 resulted in significantly unwanted impact on CCTA accuracy even when implemented by dual-source CT. Hence, based on the Asian Society of Cardiac Imaging (ASCI) published appropriateness criteria, the CCTA had an indeterminate appropriateness when the previous CACS in asymptomatic patients was ≥400 (70). 

**Table 2.** Significance and Application of “Zero Coronary Artery Calcium Score”

Study Authors	Year	Study Cohort	Results^a^
**Thompson and Stanford (59)**	2001	Not Applicable	Exclusion of significant CAD likelihood in CACS=0
**Shemesh et al. (58)**	2003	50	Low CACS characterized patients with acute coronary events
**Knez et al. (60)**	2004	2,115	Very accurate in obstructive CAD exclusion in subjects > 50 years old
**Church et al. (63)**	2007	10,746	Very low CAD risk in the intermediate term
**Akram et al. (71)**	2008	210	CACS is better in asymptomatic subjects, especially in patients < 45 years to exclude obstructive CAD
**Cademartiri et al. (72)**	2010	279	Prevalence of significant CAD was not negligible in asymptomatic patients with CACS=0
**Ergun et al. (61)**	2010	883	CACS=0 patients had positive CTA findings, especially when risk factors exist
**Gottlieb et al. (73)**	2010	291	Frequent occurrence of total coronary occlusion in CACS=0 patients
**Uretsky et al. (62)**	2011	1,119	CACS=0 is rarely accompanied by hemodynamically significant CAD
**Esteves et al. (74)**	2011	206	CACS=0 excluded inducible ischemia in an intermediate risk group
**Sonowski et al. (75)**	2011	166	Relatively low incidence of significant coronary stenosis in CACS=0 patients
**Alqarqaz et al. (76)**	2011	333	Nearly one in five patients with CACS=0 had non-calcified plaque
**Villines et al. (77)**	2011	5,128	In symptomatic patients with a CACS=0, obstructive CAD is possible and is associated with increased cardiovascular events
**Chen et al. (78)**	2012	519	Plaques are present in a significant proportion of individuals with CACS=0
**Morita et al. (79)**	2012	2,160	If patients are male and elderly even if CACS=0 the likelihood of vulnerable plaque exists especially in the presence of spotty calcification
**Kim et al. (80)**	2012	1,114	Prevalence of obstructive CAD and adverse cardiac events are not negligible in symptomatic patients with CACS=0
**Meyer et al. (81)**	2012	121	Significant CAD is extremely unlikely in symptomatic Caucasian patients with an intermediate risk score and CACS=0
**Büyükterzi et al. (82)**	2013	288	The frequency of non-calcified plaques is too high to be ignored in CACS=0
**Cho et al. (83)**	2013	4,491	A future risk of exclusive non-calcified plaque in asymptomatic subjects with CACS=0 was negligible
**Lee et al. (84)**	2013	6,531	In asymptomatic subjects with CACS=0 presence of non-calcified plaque was associated with cardiac events
**Mouden et al. (85)**	2013	868	A CACS=0 in stable patients at low or intermediate risk excludes flow-limiting CAD

^a^Abbreviations: CAD: Coronary artery disease; CACS: Coronary artery calcium score; CTA: Computed tomography angiograph

The appropriateness criteria for CCTA published in 2010 by a joint group of some American scientific societies suggested that in symptomatic patients two groups may be considered: a- in case of a calcium coverage score >400, the diagnostic influence of CACS on the decision to carry out CCTA was “uncertain” and b- in a calcium coverage score ≤400, the corresponding diagnostic effect of CACS was considered to be “appropriate” (86). Recently, Otton et al showed that in those individuals who have a CACS > 600, a negative CTCA implied an excellent short-term outcome and appeared to exclude clinically significant coronary disease (87). Ahn et al. (88) in a group of 253 patients who had CACS of > 400 found that despite good overall diagnostic accuracy, CCTA was limited by low specificity. Another study showed that the accuracy of CCTA in the presence of a high coronary calcium score may be underestimated (89).

Considering the CACS validity, other clinical applications have been introduced, as well. Over the past years, CACS has been used as standard of reference quantitative technique for diagnosis of atherosclerosis of either coronary arteries or other non-coronary arterial structures. Indeed, calcium scoring methods are also used to calculate the calcification in other body organs, including the cardiac valves like the aortic valve, in a quantitative manner (90).

The CACS was used as a reference for CAD risk. Based on the Rotterdam study, the calcium score might be utilized in re-stratification of elderly who are at intermediate risk in 10-year Framingham score to assign them in high-risk or low-risk clusters with cut-off CACS of 615 and 50 AS, respectively (91). Based on MESA database, Sirineni and coworkers proposed “coronary age” to predict CAD likelihood, formulating it based on ethnic groups and gender, so that AS was applied as an input factor to calculate the “coronary age” of any individual (92).

Not only the CACS has established as a CAD screening test, but also the coronary CTA has significantly improved to determine whether or not the coronary arterial lumen is patent. The CCTA is of more important role than CACS for CAD assessment; therefore, following CACS, patients may undergo CCTA to assess CAD likelihood. Hence, CACS has been considered to be a “gatekeeper” for CCTA (68-70, 86). Colletti et al. (93) in a group of elderly asymptomatic subjects found a remarkable relationship between CACS and forthcoming regional left ventricular wall motion abnormality as another indicator of a likely subclinical ischemic heart disease depicted in cardiac magnetic resonance imaging (MRI). Stolzmann and colleagues proposed a combination of CACS and cardiac MRI while evaluating CAD. By addition of CACS to the cardiac examination protocol, the accuracy of MRI was remarkably enhanced (94). Consecutive follow-up scorings can provide data concerning coronary calcification progression (95). Wong and colleagues (96) measuring the volume score progression and correlating that with lipid profile change assessed the efficiency of calcium scoring to evaluate the impact of lipid-lowering treatments and demonstrated that higher HDL cholesterol level was accompanied by less progression of volume score; nevertheless, could not find any evidence in favor of that CACS progression implied the low-density lipoprotein (LDL) change. Currently, many experts believe that CACS changes over the time can be considered as a method for watching the impact of lipid-lowering treatments.

Tong et al. (97) demonstrated that calcium score and left ventricular hypertrophy extent were significantly correlated in young to middle-aged African-American individuals. Women in postmenopausal age are more prone to atherosclerotic changes and CAD than premenopausal women and estrogenic hormones used to treat postmenopausal syndrome result in reduction of coronary atherosclerosis. Long term hormone replacement therapy has been shown to be effective on CACS as an indicator of CAD risk and those postmenopausal individuals utilizing estrogen for minimum 10 years revealed remarkably less CACS in comparison with those who had shorter period uses (98, 99). It has been shown that visceral and subcutaneous fats have different effects on cardiovascular risks. Pericardial fat is one of the visceral fat structures and as shown by Yun et al. (100) has an independent character in coronary calcification regardless of anthropometric measurements. In another study, Sabour and colleagues pointed out that there is a relationship between persistent abdominal obesity with high CACS, which suggests an increased risk of coronary atherosclerosis (101). Nonetheless, Shabestari and colleagues (102) have displayed that anthropometric measurements are generally more reliable than ultrasonic abdominal fat measurements in prediction of CAD. Jung et al. (103) recommended measuring the CACS for further coronary atherosclerosis assessment in cases of fatty liver and increased level of alanine aminotransferase. The ascorbic acid is a crucial antioxidant. It has been proposed that there is an association between ascorbic acid deficiency and cardiovascular disease risk (104) and hence, a study by Simon et al was performed correlating plasma ascorbic acid level and calcium score confirming their substantial correlation of a group of young men. However, this was not detected in a corresponding young female group (105, 106).

## 4. Conclusions

As a conclusion it should be reminded that based on many data gathered over recent decades and despite presence of some controversies, the CACS advantages and disadvantages are appropriately evaluated and the correct form is its and not it's. clinical and research applications are accepted. These have led to general acceptance of calcium scoring as a standard of reference for determination of risk of cardiac events. In spite of presence of controversies, CACS has gained an acceptance to be an indicator of cardiac events risk and likely will even be more persuasive in further cardiovascular risk management in future.
